# Substance and Internet use during the COVID-19 pandemic in China

**DOI:** 10.1038/s41398-021-01614-1

**Published:** 2021-09-23

**Authors:** Qiuping Huang, Xinxin Chen, Shucai Huang, Tianli Shao, Zhenjiang Liao, Shuhong Lin, Yifan Li, Jing Qi, Yi Cai, Hongxian Shen

**Affiliations:** 1 National Clinical Research Center for Mental Disorders, and Department of Psychiatry, the Second Xiangya Hospital of Central South University, Changsha, Hunan China; 2 Institute of Mental Health of Central South University, Chinese National Technology Institute on Mental Disorders, Hunan Key Laboratory of Psychiatry and Mental Health, Hunan Medical Center for Mental Health, Changsha, Hunan China; 3 Department of Psychiatry, the Fourth People’s Hospital of Wuhu, Wuhu, Anhui China; 4 Department of Psychiatry, Comorbid Somatic Diseases, Kangning Hospital of Shenzhen, Shenzhen, Guangdong China; 5 Department of Psychiatry, Brain Hospital of Hunan Province, Changsha, Hunan China

**Keywords:** Human behaviour, Addiction

## Abstract

The coronavirus disease 2019 (COVID-19) has adversely influenced human physical and mental health, including emotional disorders and addictions. This study examined substance and Internet use behavior and their associations with anxiety and depression during the COVID-19 pandemic. An online self-report questionnaire was administered to 2196 Chinese adults between February 17 and 29, 2020. The questionnaire contained the seven-item Generalized Anxiety Disorder Scale (GAD-7) and Patient Health Questionnaire (PHQ-9), questions on demographic information, and items about substance and Internet use characteristics. Our results revealed that males consumed less alcohol (*p* < 0.001) and areca-nut (*p* = 0.012) during the pandemic than before the pandemic. Age, gender, education status, and occupation significantly differed among increased substance users, regular substance users, and nonsubstance users. Time spent on the Internet was significantly longer during the pandemic (*p* < 0.001) and 72% of participants reported increased dependence on the Internet. Compared to regular Internet users, increased users were more likely to be younger and female. Multiple logistic regression analysis revealed that age <33 years (OR = 2.034, *p* < 0.001), increased substance use (OR = 3.439, *p* < 0.001), and increased Internet use (OR = 1.914, *p* < 0.001) were significantly associated with depression. Moreover, anxiety was significantly related to female gender (OR = 2.065, *p* < 0.001), “unmarried” status (OR = 1.480, *p* = 0.017), nonstudents (OR = 1.946–3.030, *p* = 0.001), and increased substance use (OR = 4.291, *p* < 0.001). Although there was a significant decrease in social substance use during the pandemic, more attention should be paid to increased Internet use. Increased Internet use was significantly associated with both anxiety and depression, and increased substance use was related to depression. Professional support should be provided to vulnerable individuals to prevent addiction.

## Introduction

Since December 2019, COVID-19 has emerged and spread rapidly across the globe. The World Health Organization declared COVID-19 a pandemic on March 11, 2020 [1]. Due to concerns about infection and stigma toward infected individuals and their families, as well as occupational factors, economy, isolation, loneliness, and other factors [2, 3], the prevalence of various psychological disorders—such as anxiety, depression, stress, and insomnia—has increased [4–6]. To cope with these altered circumstances, people are more likely to indulge in addictive behaviors (including substance use and behavioral addictions) and/or relapse from abstinence [7–9]. Previous research has confirmed that major disasters have a significant influence on addictive behaviors, such as smoking, drinking, and Internet use [10–12].

Substance use and COVID-19 are closely related. Individuals with substance use have an increased risk of COVID-19 infection and of developing severe symptoms [13]. Smoking suppresses respiratory functions and may increase the risk of illness and even death among smokers, irrespective of whether they use tobacco, cannabis, or heroin [14]. One review found that cigarette smoking causes immune defects, such as damaging proinflammatory and immunosuppressive factors [15], making smokers particularly vulnerable to COVID-19. On the contrary, COVID-19 increases the risk of substance dependence. An increase in substance use as an emotional coping strategy was linked to the pandemic and its subsequent psychological distress, such as depression and posttraumatic stress disorder [16]. Furthermore, there are limited medical resources for these groups, as most available resources are being expended on the COVID-19 pandemic [17]. Moreover, as numerous shops, restaurants, and bars were closed, it was more difficult for users to obtain these substances. A brief Chinese report showed that some regular smokers and drinkers increased their substance use during the pandemic, and relapses from abstinence were relatively common [18]. However, another study showed that alcohol consumption in China during the pandemic was relatively low [19]. As such, it remains unclear whether there has been a change in substance use in China, and how the change occurs, if there has.

During the pandemic, to slow down the spread of the virus, people were advised to stay at home, and outdoor social activities were drastically reduced. Empirical evidence suggests increased consumption of online entertainment. An online survey of the general population in China showed that during the pandemic, 46.8% of participants reported increased dependence on Internet use, while 16.6% reported longer Internet use duration [18]. Another study in Japan showed that treatment seekers with excessive gaming or Internet use reported significantly more daily hours spent on the Internet, smartphones, gaming, and video viewing due to the stay-at-home measure [20]. Although the stay-at-home measure and Internet use could facilitate the prevention of further infection spread and control the pandemic, overuse of the Internet and gaming can lead to behavioral addiction.

Psychological distress has become a significant public health issue during the pandemic and has attracted the attention of researchers and the public alike. A nationwide multicenter cross-sectional study on 19372 participants in China reported that 11–13.3% had anxiety, depressive, or insomnia symptoms, and these were associated with working as frontline medical staff, living in Hubei Province, having close contact with patients with COVID-19, being 35–49 years old, and not engaging in outdoor activities for 2 weeks [4]. It is known that addictive behaviors are related to anxiety and depression [21, 22]. However, few studies reported on these relationships during the pandemic.

Due to the interactive impact of the pandemic on substance use, the changing trend of substance use deserves further clarification. Most studies have focused on gaming or certain specific Internet use behavior during the pandemic, but few have comprehensively investigated the full spectrum of Internet use behavior. Few studies have reported on the relationship between addiction behavior and emotions during the pandemic. This study examined substance and Internet use behavior and their associations with anxiety and depression during the COVID-19 pandemic. We hypothesized that there has been a decrease in substance use and an increase in Internet use during the pandemic, and that increases both in substance use and Internet use are related to anxiety and depression.

## Methods

### Participants

The present study was an anonymous online survey of the general population in China. It was conducted from February 17 to 29, 2020. Participants were recruited through the widely used Chinese questionnaire survey website, “Questionnaire Star Platform.” The QR code and link to the survey were shared in group chats and in the “Moments” section of the authors’ WeChat. Participants were instructed to complete the questionnaire online. At the beginning of the web page, a brief introduction was presented that included a detailed background, purposes of the study, and study procedures. Individuals who provided consent for participation and met the following criteria were recruited: of Chinese nationality, at least 18 years old, living in China, and Chinese speaker. Individuals who were infected with COVID-19 and those who did not complete the questionnaire were excluded.

The Ethics Committee of the Second Xiangya Hospital of Central South University approved the study protocol. All participants answered a yes–no question to indicate their voluntary participation in the study prior to completing the questionnaire.

### Measures

The questionnaire consisted of four parts: demographics, substance use, Internet use, and emotion (depression and anxiety). Demographic variables included gender, age, education, occupation, marital status, and residence (Hubei vs. other provinces of China).

#### Substance use

Participants provided information concerning their use of alcohol, tobacco, sedative-hypnotic, areca nut, illicit drugs (heroin, methamphetamine, ketamine, cocaine, and cannabis), and other substances. Example questions were asked: “One month before the COVID-19 outbreak, which of the following substances did you use?” and “After the COVID-19 outbreak, which of the following substances did you use?” Answer options included “none,” “alcohol,” “tobacco,” “sedative-hypnotic,” “areca nut,” “heroin,” “methamphetamine,” “ketamine,” “cocaine,” “cannabis,” and “others” (as specified by the responders). Individuals should choose one or more options. Participants were also asked, “How did your main substance use behavior change after the COVID-19 outbreak?” Responses were scored on a 5-point scale: 1 = using a lot more, 2 = using a little more, 3 = no change, 4 = using a little less, and 5 = using much less. Participants who reported using “a lot more” or a “little more” since the outbreak were considered increased substance users. Participants who reported “no change” or “using less” were considered regular substance users, while participants who reported no substance use were considered non-drug users [23, 24].

#### Internet use

The following questions were asked to obtain information about Internet use: “How many hours did you spend on the Internet every day before and after the COVID-19 outbreak?” and “What was your main Internet use behavior before and after the COVID-19 outbreak?” Answer options included “Internet gaming,” “short videos,” “films and television,” “network novels,” “shopping online,” “browsing health information,” and “others” (as specified by the responders). Participants were then asked, “How did your main Internet use behavior change after the COVID-19 outbreak?” Responses were scored on a 5-point scale: 1= using a lot more, 2 = using a little more, 3 = no change, 4 = using a little less, and 5 = using much less. Participants who reported using a lot more or a little more Internet after the outbreak were considered increased Internet users, while participants reporting no or little change in Internet use were considered regular Internet users.

#### Anxiety

The 7-item Generalized Anxiety Disorder Scale (GAD-7) was used to evaluate the presence and severity of general anxiety [25]. All items are rated on a 4-point rating scale (0 = not at all to 3 = nearly every day), and the total score ranges from 0 to 21, with higher scores indicating more severe anxiety. A total score of 10 or more indicates positive results in China [26]. The Chinese GAD-7 has good reliability and validity in assessing anxiety symptoms in the Chinese population [26, 27]. In this study, the Cronbach’s α coefficient for the GAD-7 was 0.926.

#### Depression

The presence of depression in the past 2 weeks was assessed using the Chinese version of the Patient Health Questionnaire (PHQ-9) [28]. All items of the PHQ-9 are rated on a four-point scale, ranging from 0 (never) to 3 (nearly every day). The total score ranges from 0 to 27, with higher scores indicating greater severity of depressive symptoms. A cutoff score of 10 or more indicates the presence of depression [29]. The Chinese version of the PHQ-9 has been proved to be reliable and valid for assessing depression in the general population [29, 30]. In the present study, the Cronbach’s α coefficient of PHQ-9 was 0.904.

### Statistical analysis

Self-reported substance and Internet use characteristics were described and compared by performing a chi-square (*χ*^2^) test. Differences in demographic characteristics among groups were compared using a chi-square (*χ*^2^) test, and differences in online time and PHQ-9 and GAD-7 scores among subgroups were examined by performing Wilcoxon rank-sum and Kruskal-Wallis H tests. To test whether increased addiction behavior was significantly associated with anxiety and depression, a multiple logistic regression with a backward stepwise method was used, which included depression and anxiety as predictors, and all significant factors in the univariate analysis as covariates. Odds ratios (ORs) and 95% confidence intervals (CIs) were generated for each variable. The significance level was set at *P* < 0.05 (two-sided). To adjust for the multiple post hoc tests, the significance level was set at *P* < 0.01 (two-sided). All statistical analyses were conducted using the SPSS 22.0.

## Results

A total of 2230 participants completed the questionnaire. Seven individuals infected with COVID-19 and 27 living abroad were excluded. The final sample size was 2196. The mean age of the participants was 34.26 (standard deviation [SD]:11.78, range: 18–74), 1445 (65.8%) were women, and 89 (4.1%) were Hubei residents.

Among these 2196 participants, 14.1% reported consuming alcohol during the pandemic, which was significantly lower than the rate of 16.1% before the pandemic (*p* < 0.001); 3.2% reported that they used areca nut during the pandemic, which was significantly lower than the 3.9% reported before (*p* = 0.004). As shown in Table 1, these significant decreases were observed in males (*p* < 0.001 for alcohol and *p* = 0.012 for areca-nut). There was no significant difference in the rates of tobacco use, sedative-hypnotics, and other substances used before and during the pandemic (Table 1). In total, 5.7% of participants reported increased substance use during the pandemic, 22.5% reported no increase, and 71.8% reported no substance use. We found that age, gender, education status, and occupation differed significantly among increased substance users, regular substance users, and non-substance users. However, there were no significant differences in the rate of marital status and place of residence among the three groups. Detailed multiple post hoc test results are presented in Table 2.

**Table 1:** Substance use and comparison between pre-pandemic and during the pandemic by each substance.

	Male (751, 34.2%)			Female (1445, 65.8%)			Total (2196, 100%)		
	Before	During	*p*	Before	During	*p*	Before	During	*p*
Tobacco	246 (32.8)	238 (31.7)	0.229	27 (1.9)	23 (1.6)	0.424	273 (12.4)	261 (11.9)	0.111
Alcohol	262 (34.9)	229 (30.5)	<0.001	92 (6.4)	80 (5.5)	0.162	354 (16.1)	309 (14.1)	<0.001
Sedative hypnotic	18 (2.4)	13 (1.7)	0.227	32 (2.2)	28 (1.9)	0.424	50 (2.3)	41 (1.9)	0.108
Areca-nut	71 (9.5)	59 (7.9)	0.012	14 (1.0)	11 (0.8)	0.375	85 (3.9)	70 (3.2)	0.004
Illicit drugs^a^	4 (0.5)	4 (0.5)	1.000	4 (0.3)	1 (0.1)	0.250	8 (0.4)	5 (0.2)	0.453
Others^b^	9 (1.2)	12 (1.6)	0.453	23 (1.6)	21 (1.5)	0.804	32 (1.5)	33 (1.5)	1.000
None	385 (51.3)	400 (53.3)	0.105	1304 (90.2)	1317 (91.1)	0.154	1689 (76.9)	1717 (78.2)	0.025

^a^Illicit drugs include heroin, methamphetamine, ketamine, cocaine, and cannabis.

^b^Others include café, oryzanol, and any other substances participants did not disclose.

**Table 2:** Comparison among different types of substance use by socio-demographic variables.

Characteristics	Increased substance users (*n* = 125)	Regular substance users (*n* = 495)	Non-substance users (*n* = 1576)	*χ*^2^	*p*	Paired comparisons
Age						
<33	54 (43.2)	208 (42.0)	834 (52.9)	20.283	<0.001	3^*^
≥33	71 (56.8)	287 (58.0)	742 (47.1)			
Gender						
Male	86 (68.8)	260 (52.5)	405 (25.7)	190.993	<0.001	1^*^;2^*^;3^*^
Female	39 (31.2)	235 (47.5)	1171 (74.3)			
Education						
High school and below	26 (20.8)	128 (25.9)	304 (19.3)	9.849	0.007	3^*^
College and above	99 (79.2)	367 (74.1)	1272 (80.7)			
Occupation						
Physical labor	43 (34.4)	112 (22.6)	392 (24.9)	28.958	<0.001	2^*^;3^*^
Mental labor	41 (32.8)	145 (29.3)	377 (23.9)			
Students	33 (26.4)	183 (37.0)	685 (53.5)			
Unemployed	8 (6.4)	55 (11.1)	122 (7.7)			
Marital status						
Married	53 (42.4)	193 (39.0)	639 (40.5)	0.622	0.733	
Unmarried	72 (57.6)	302 (61.0)	937 (59.5)			
Place of residence						
Hubei	10 (8.0)	19 (3.8)	60 (3.8)	5.312	0.070	
Other places	115 (92.0)	476 (96.2)	1516 (96.2)			

1: Increased Substance Users vs. Regular Substance Users.

2: Increased Substance Users vs. Nonsubstance Users.

3: Regular Substance Users vs. Nonsubstance Users.

^*^p < 0.01.

Time spent on the Internet during the pandemic was found to be significantly longer than before (during vs. before: 5.4 ± 3.2 vs. 3.4 ± 2.4; t = 39.359; *p* < 0.001). Before the COVID-19 outbreak, the top four Internet use behaviors were films and television (21.4%), short videos (15.5%), shopping online (12.9%), and Internet gaming (12.3%). In contrast, during the pandemic, the top four Internet use behaviors were browsing health information (30.3%), films and television (20.6%), short videos (14.7%), and Internet gaming (12.8%). In addition, only browsing health information and Internet gaming use showed increases in percentages among all Internet use behaviors. More details are presented in Table 3. In total, 72% of participants reported an increased dependence on Internet use during the pandemic. Compared to regular Internet users, increased users were more likely to be younger and female (see Table 4).

**Table 3:** Distribution of the main Internet behaviors before and during the pandemic.

Internet behaviors	Main use	
	Before	During
Internet gaming	270 (12.3)	282 (12.8)
Short videos	341 (15.5)	322 (14.7)
Films and television	469 (21.4)	453 (20.6)
Network novels	185 (8.4)	154 (7.0)
Shopping online	284 (12.9)	66 (3.0)
Browsing health information	249 (11.3)	666 (30.3)
Others^a^	398 (18.1)	253 (11.5)

^a^Others include Weibo, Zhihu, working online, and any other Internet behaviors participants did not disclose.

**Table 4:** Comparison between increased and regular Internet users by socio-demographic variables.

Characteristics	Increased Internet users (*n* = 1581)	Regular Internet users (*n* = 615)	*χ*^2^	*p*
Age				
<33	813 (51.4)	283 (46.0)	5.178	0.023
≥33	768 (48.6)	332 (54.0)		
Gender				
Male	517 (32.7)	234 (38.0)	5.627	0.018
Female	1064 (67.3)	381 (62.0)		
Education				
High school and below	317 (20.1)	141 (22.9)	2.219	0.136
College and above	1264 (79.9)	474 (77.1)		
Occupation				
Physical labor	396 (25.0)	151 (24.6)	1.987	0.575
Metal labor	396 (25.0)	167 (27.2)		
Students	660 (41.7)	241 (39.2)		
Unemployed	129 (8.2)	56 (9.1)		
Marital status				
Married	691 (42.4)	249 (39.5)	1.567	0.213
Unmarried	938 (57.6)	381 (60.5)		
Place of residence				
Hubei	63 (4.0)	26 (4.2)	0.067	0.796
Other places	1518 (96.0)	589 (95.8)		

A total of 278 (12.7%) participants had depression (PHQ-9 score ≥ 10), and 190 (8.7%) had anxiety (GAD-7 score ≥ 10). The increased substance users’ scores on the PHQ-9 and GAD-7 were significantly higher than those of regular and non-users (increased substance users vs. regular users vs. non-users, PHQ-9: 7.6 ± 6.9 vs. 4.1 ± 5.4 vs. 4.0 ± 5.0, z = 45.017, p < 0.001; GAD-7: 6.6 ± 6.2 vs. 3.2 ± 4.5 vs. 3.0 ± 4.2, *z* = 58.884, *p* < 0.001). The increased Internet users’ scores on the PHQ-9 and GAD-7 were significantly higher than those of regular users (increased users vs. regular users, PHQ-9: 4.7 ± 5.4 vs. 2.9 ± 4.7, *z* = 10.697, *p* < 0.001; GAD-7: 3.6 ± 4.6 vs. 2.3 ± 4.0, *z* = 7.791, *p* < 0.001).

Results from the chi-square tests (see Table 5) showed that individuals with depression were more likely to be young (*p* < 0.001) and have increased substance (*p* < 0.001) and Internet use (*p* < 0.001). Anxiety was more prevalent among females (*p* = 0.038), nonstudents (*p* < 0.001), unmarried persons (*p* = 0.024), and increased substance users (*p* < 0.001) (see Table 6). Multiple logistic regression analysis (see Table 7) revealed that three variables were still significantly associated with depression: age <33 years (OR = 2.034, *p* < 0.001), increased substance use (OR = 3.439, *p* < 0.001), and increased Internet use (OR = 1.914, *p* < 0.001). Further, anxiety was significantly related to female gender (OR = 2.065, *p* < 0.001), “unmarried” status (OR = 1.480, *p* = 0.017), nonstudents (OR = 1.946–3.030, *p* ≤ 0.001), and increased substance use (OR = 4.291, *p* < 0.001) (Table 5).

**Table 5:** Socio-demographic, substance, and Internet use characteristics and comparison between positive and negative depression by variables.

Characteristics	Depression positive (*n* = 278)	Depression negative (*n* = 1918)	*χ*^2^	*p*
Age				
<33	179 (64.4)	917 (47.8)	26.693	<0.001
≥33	99 (35.6)	1001 (52.2)		
Gender				
Male	83 (29.9)	668 (34.8)	2.667	0.102
Female	195 (70.1)	1250 (65.2)		
Education				
High school and below	68 (24.5)	390 (20.3)	2.505	0.113
College and above	210 (75.5)	1528 (79.7)		
Occupation				
Physical labor	80 (28.8)	467 (24,3)	4.948	0.176
Mental labor	70 (25.2)	493 (25.7)		
Students	100 (36.0)	801 (41.8)		
Unemployed	28 (10.1)	157 (8.2)		
Marital status				
Married	123 (44.2)	762 (39.7)	2.058	0.151
Unmarried	155 (55.8)	1156 (60.3)		
Place of residence				
Hubei	17 (6.1)	72 (3.8)	3.481	0.062
Other places	261 (93.9)	1846 (96.2)		
Substance use				
Increased users	38 (13.7)	87 (4.5)	37.732	<0.001
Regular users	57 (20.5)	438 (22.8)		
Non-users	183 (65.8)	1393 (72.6)		
Internet use				
Increased users	231 (83.1)	1350 (70.4)	19.447	<0.001
Regular users	47 (16.9)	568 (29.6)

**Table 6:** Sociodemographic, substance and Internet use characteristics and comparison between positive and negative anxiety by variables.

Characteristics	Anxiety positive (*n* = 190)	Anxiety negative (*n* = 2006)	*χ*^2^	*p*
Age				
<33	85 (44.7)	1011 (50.4)	2.226	0.136
≥33	105 (55.3)	995 (49.6)		
Gender				
Male	52 (27.4)	699 (34.8)	4.312	0.038
Female	138 (72.6)	1307 (65.2)		
Education				
High school and below	42 (22.1)	416 (20.7)	0.197	0.657
College and above	148 (77.9)	1590 (79.3)		
Occupation				
Physical labor	61 (32.1)	486 (24.2)	24.645	<0.001
Mental labor	55 (28.9)	508 (25.3)		
Students	48 (25.3)	853 (42.5)		
Unemployed	26 (13.7)	159 (7.9)		
Marital status				
Married	62 (32.6)	823 (41.0)	5.084	0.024
Unmarried	128 (67.4)	1183 (59.0)		
Place of residence				
Hubei	12 (6.3)	77 (3.8)	2.739	0.098
Other places	178 (93.7)	1929 (96.2)		
Substance use				
Increased users	27 (14.2)	98(4.9)	28.969	<0.001
Regular users	44 (23.2)	451 (22.5)		
Non-users	119 (62.6)	1457 (72.6)		
Internet use				
Increased users	147 (77.4)	1434 (71.5)	2.979	0.084
Regular users	43 (22.6)	572 (28.5)

**Table 7:** Results of multiple logistic regression analysis on factors significantly associated with depression and anxiety.

Variables	*p*	OR (95%CI)
**Depression**		
Age(<33 vs. ≥33)	<0.001	2.034 (1.558, 2.656)
Substance use (regular users vs. nonusers)	0.493	1.119 (0.811, 1.543)
Substance use (increased users vs. nonusers)	<0.001	3.439 (2.257, 5.238)
Internet use (increased users vs. regular users)	<0.001	1.914 (1.371, 2.670)
**Anxiety**		
Gender (female vs. male)	<0.001	2.065 (1.433, 2.977)
Material status (unmarried vs. married)	0.017	1.480 (1.072, 2.043)
Occupation (physical labor with students)	<0.001	2.334 (1.560, 3.494)
Occupation (mental labor with students)	0.001	1.946 (1.293, 2.930)
Occupation (unemployed with students)	<0.001	3.030 (1.815, 5.059)
Substance use (regular users vs. nonusers)	0.095	1.379 (0.946, 2.012)
Substance use (increased users vs. nonusers)	<0.001	4.291 (2.595, 7.097)

## Discussion

To our knowledge, this is the first study to examine both substance and Internet use behaviors, and their associations with anxiety and depression during the COVID-19 pandemic. Overall, we observed a decrease in substance use and an increase in Internet use. Significant relationships were observed between depression and Internet and substance use, as well as between anxiety and substance use during the pandemic.

As a stressful event, the COVID‐19 pandemic and its associated quarantine measures have adversely affected substance dependence [31]. However, in the present study, participants reported a decreased use of several substances (alcohol, areca-nut), while the use of others remained relatively stable during COVID-19, compared to the pre-pandemic period. This result is consistent with Wang et al.’s study [19] but inconsistent with other studies that showed marginally increased alcohol and cigarette consumption [18, 31]. However, there are no reports on changes in areca nut consumption—a socially used substance like alcohol and tobacco in China. The Chinese government took nationwide measures to restrict outdoor activities following the Spring Festival to reduce COVID-19 spread. Social celebrations and parties were strictly prohibited, which may have reduced social substance use. Potential explanations include lower craving levels compared to the pre-pandemic due to limited availability, and the prompted healthy behavior due to lockdown [32, 33]. In contrast, restricted socialization also reduced social pressure, as substance users were often stigmatized and marginalized [13, 14, 34]. Although decreased consumption was observed in males in this study, it also found that males are at particular risk of substance use, warranting more attention for this group [19]. This is in line with a previous study that reported that men were more likely to report substance use and cope with stress by increasing substance use [35].

Consistent with the findings of previous studies, the results showed an increase in Internet use [18, 36]. There are several explanations for this finding. First, as most outdoor activities were restricted, consumption of online entertainment inevitably increased. Second, during the pandemic, people had to stay at home and work and study, as well as engage in essential social interactions on the Internet which naturally extended their duration of Internet use. Third, Internet use is a strategy to cope with stressful life conditions [37]. Compared with other potential behaviors, such as substance use and overeating, Internet use is a less harmful coping strategy [38, 39]. In this study, more people spent time on films and television, short videos, and playing games. However, one possible consequence of this might be a general increase in Internet dependence, especially among the young generation, and difficulties in reintegration after the pandemic [40, 41]. Therefore, it is essential to promote balanced Internet use behavior during the COVID-19 pandemic.

In this study, participants reporting increased Internet use were likely to be younger and female. The young generation is particularly vulnerable to Internet addiction, especially given the impact of the pandemic [34, 42, 43]. Particular attention should be paid to the prevention of behavioral addiction in young people. Although Internet addiction has always been considered male-dominant [44, 45], females are also vulnerable [46–49]. Based on previous findings, it can be supposed that females might be more susceptible to several types of Internet behaviors, including the use of social networking sites and online shopping [47, 50]. During the pandemic, a considerable increase was shown in the use of social networks, which might explain the gender difference in increasing Internet use. Moreover, the percentage of browsing medical information and Internet gaming increased during this period. On the one hand, it is necessary to issue authorial and scientific information about COVID-19 to avoid misleading the public. On the other hand, individuals should try to discriminate against misleading facts and rumors about COVID-19 and shift their attention from the Internet to other activities, such as sports, music, and cooking [18, 51].

The two-week prevalence of depression (12.7%) and anxiety (8.7%) among the general population in the present study was lower than those reported in previous studies (depression: 22.1–43.7%; anxiety: 21.6–49.6%) [52–56]. The prevalence disparities might be related to the different cutoff values of the PHQ-9 and GAD-7 used in previous studies, the gender ratio, the specific periods of the pandemic, and the proportion of Hubei residents in the study sample. Our results confirmed significant associations between increased addictive behaviors and anxiety and depression after adjusting for potential confounders during the COVID-19 pandemic. Previous studies on the impact of disasters reported increased substance use due to an increase in psychological stress [24, 57], which was associated with Internet addiction [58, 59]. Evidence has shown that substance use is a coping strategy employed after disasters [60, 61]. Substance and Internet use may be common ways to mitigate negative emotions and stress. The general population is encouraged to maintain an ordinary life under safe conditions and use whatever available psychosocial service system is present to cope with this stress [62]. More attention should be paid to groups that are at high risk for addiction, as excessive substance and Internet use may also lead to psychological distress during the pandemic [51, 63]. As such, more studies should examine the causal relationship between addictive behaviors and emotional problems.

There are several limitations to this study. First, as a cross-sectional survey, it could not determine the causal relationship between increased addiction behavior and mental health indicators. Second, self-reported changes in addiction behavior are arguably unstandardized and may be an unreliable and insufficient measure of the construct. Furthermore, pre-pandemic information may entail recall bias. Finally, the convenience sampling method and the limited sample representativeness limit the generalizability of our findings. Larger scale and longitudinal cohort studies should be conducted to investigate addictive behaviors during and after the pandemic.

## Conclusions

This study investigated changes in substance and Internet use during the COVID-19 pandemic. Our findings provide evidence of a significant association between increased addictive behaviors and psychological problems (such as depression and anxiety) during the pandemic. At this stage, addiction-related issues should be considered. Therefore, it is necessary to establish proper measures and support services for such vulnerable populations. The results also represent a preliminary step toward further understanding the relationship between increased addictive behaviors and potentially related factors (gender and age) during the pandemic. Nevertheless, due to the cross-sectional design and limited generalizability of this study, more studies are warranted to investigate these associations.
